# Chronic unpredictable mild stress alters odor hedonics and adult olfactory neurogenesis in mice

**DOI:** 10.3389/fnins.2023.1224941

**Published:** 2023-08-03

**Authors:** Anna Athanassi, Marine Breton, Laura Chalençon, Jérome Brunelin, Anne Didier, Kevin Bath, Nathalie Mandairon

**Affiliations:** ^1^INSERM, U1028, CNRS UMR5292, Neuropop Team, Lyon Neuroscience Research Center, Université Claude Bernard Lyon 1, Université Jean Monnet, Bron, France; ^2^Centre Hospitalier Le Vinatier, Bron, France; ^3^INSERM, U1028, CNRS UMR5292, PSYR2 Team, Lyon Neuroscience Research Center, Université Claude Bernard Lyon 1, Université Jean Monnet, Bron, France; ^4^Division of Developmental Neuroscience, New York State Psychiatric Institute, Research Foundation for Mental Hygiene, New York, NY, United States; ^5^Department of Psychiatry, Columbia University Medical College, New York, NY, United States

**Keywords:** olfactory bulb, odor hedonics, adult neurogenesis, chronic mild stress, emotional alteration

## Abstract

Experiencing chronic stress significantly increases the risk for depression. Depression is a complex disorder with varied symptoms across patients. However, feeling of sadness and decreased motivation, and diminished feeling of pleasure (anhedonia) appear to be core to most depressive pathology. Odorants are potent signals that serve a critical role in social interactions, avoiding danger, and consummatory behaviors. Diminished quality of olfactory function is associated with negative effects on quality of life leading to and aggravating the symptoms of depression. Odor hedonic value (I like or I dislike this smell) is a dominant feature of olfaction and guides approach or avoidance behavior of the odor source. The neural representation of the hedonic value of odorants is carried by the granule cells in the olfactory bulb, which functions to modulate the cortical relay of olfactory information. The granule cells of the olfactory bulb and those of the dentate gyrus are the two major populations of cells in the adult brain with continued neurogenesis into adulthood. In hippocampus, decreased neurogenesis has been linked to development or maintenance of depression symptoms. Here, we hypothesize that chronic mild stress can alter olfactory hedonics through effects on the olfactory bulb neurogenesis, contributing to the broader anhedonia phenotype in stress-associated depression. To test this, mice were subjected to chronic unpredictable mild stress and then tested on measures of depressive-like behaviors, odor hedonics, and measures of olfactory neurogenesis. Chronic unpredictable mild stress led to a selective effect on odor hedonics, diminishing attraction to pleasant but not unpleasant odorants, an effect that was accompanied by a specific decrease in adult neurogenesis and of the percentage of adult-born cells responding to pleasant odorants in the olfactory bulb.

## Introduction

Depressive symptoms can be complex and vary widely between individuals as well as within individuals with recurrent episodes. In major depressive disorder (MDD), core symptoms include anhedonia and diminished motivation. In humans, depression is often also associated with disturbance in olfactory sensory function (Negoias et al., 2016; Croy and Hummel, 2017; Pabel et al., 2018; Athanassi et al., 2021). Like depression, olfactory sensory function is complex (including features such as familiarity, intensity, and identity) with the hedonic perception of odor dominating. Hedonic value is generally the first criterion used by humans to describe and categorized odorants (Schiffman, 1974; Richardson and Zucco, 1989; Yeshurun and Sobel, 2010) and represents a valuable source of information for decision-making and guiding goal-oriented motivated behaviors. Across species (including humans), the hedonic value of odorants is a critical driving force for food intake or social interactions supporting survival and regulation of the emotional state. Interestingly, odor hedonic value (pleasantness) is the dimension of olfactory perception most strongly affected in depression (Naudin et al., 2012). Consequently, altered odor hedonics can cause disturbances in food intake or mood which worsen depressive symptoms and contribute significantly to negative outcomes across a range of diseases with comorbid depression. The critical neural substrate influencing odor hedonics appears to be the granule cell layer of the olfactory bulb (OB), a modulator of the first cortical relay of olfactory information (Kermen et al., 2016). Optogenetic inhibition of granule cell activity modulates approach behavior to odorants in rodents, which is used to evaluate whether an odorant is pleasant or unpleasant in an animal model (Kermen et al., 2016; Midroit et al., 2021). The granule cells, significantly influence processing of hedonic information and interestingly are the largest population of cells with ongoing adult neurogenesis (Lledo and Valley, 2016). Stem cells located in the subventricular zone of the lateral ventricles give rise to neuroblasts that migrate along the rostral migratory stream to reach the OB and functionally integrate the pre-existing network (Alvarez-Buylla and Garíca-Verdugo, 2002; Carlén et al., 2002; Belluzzi et al., 2003; Carleton et al., 2003; Magavi et al., 2005). These new neurons are key elements of odor processing by shaping the activity of the relay cells (Schoppa and Urban, 2003; Lledo et al., 2005; Lledo and Saghatelyan, 2005). The other major brain structure with high rates of adult neurogenesis, is the hippocampus (Lledo et al., 2006; Kempermann, 2015). Prior work has implicated alterations in hippocampal neurogenesis on both the development and response to treatment for depression (Santarelli et al., 2003; Planchez et al., 2019). Like the hippocampus, the OB serves a significant role in cognitive processes, memory formation and retrieval, and emotional regulation (Bagur et al., 2021; Mofleh and Kocsis, 2021; Salimi et al., 2022).

Despite prior work implicating disturbance in neurogenesis in hippocampus with depression few studies have investigated the relationship between depressive-like behaviors and OB neurogenesis and olfactory processing (Lledo and Valley, 2016; Siopi et al., 2016). Chronic stress is known to increase the risk for depression, alter morphology and functioning of the hippocampus including alteration of adult neurogenesis (McEwen and Magarinos, 1997). Prior work has established that chronic unpredictable mild stress (CUMS) can reliably induce behavioral profiles in mice that resemble depressive symptoms in humans (Pothion et al., 2004; Nollet et al., 2012; van Boxelaere et al., 2017).

Here, the aim of this work was to test the impact of CUMS on olfactory hedonics in mice, its effects on adult OB neurogenesis, and the activity of adult-born neurons during hedonic valuation. In CUMS exposed and control mice, behavioral measures of both depressive-like and anxiety-like behaviors (Willner et al., 1987; Papp and Moryl, 1994; Willner, 2017) were assessed. In conjunction, the hedonic processing of olfactory signals was tested in an olfactory preference test (Kermen et al., 2016; Midroit et al., 2021). Finally, measures of adult-born neuron number and activity were measured in the OB of mice following hedonic preference testing. The CUMS protocol led to higher levels of anhedonia and increased anxiety-like behaviors compared with control mice. Importantly, the CUMS paradigm induced olfactory hedonic alteration, significantly diminishing interest in pleasant but not unpleasant odorants. At the cellular level, CUMS decreased the density of adult-born neurons in the OB as well as the percentage of adult-born neurons responding to pleasant but not unpleasant odorants.

## Materials and methods

### Animals

Twenty adult male C57Bl6/J mice (8 weeks, Charles River Laboratories, L’Arbresle, France) were used in this study. Mice were randomly assigned to one of two groups, one control group (*n* = 10; housed in groups of five in standard laboratory cages) and one stressed group (*n* = 10; placed in individual standard laboratory cages). All experiments were done in accordance with the European Community Council Directive of 22nd September 2010 (2010/63/UE) and the National Ethics Committee (Agreement APAFIS#20702_2019072614086203_v2). Mice were kept on a 12 h light/dark cycle at a constant temperature of 22°C with food and water *ad libitum* except during the stress protocol (Table 1). All efforts were made to minimize animal suffering.

**Table 1:** CUMS protocol stressors (see Supplementary Table 1 for examples of daily sequences of events).

Stimuli	Duration
Sawdust bedding removed	About 6 h
Mouse cage change	Permanent
Cage titling at 45°	About 1 h
Wet sawdust (200 mL of water for 100 g sawdust)	3 to 12 h
Nycthemeral rhythm perturbation	About 3 h through the night
Food and water deprivation	About 12 h
Mice pair housed (with or without dry or wet sawdust)	About 3 h or through the night
Mice hung by their tail	About 1 min
Change sawdust	Twice (each hour)

### Chronic unpredictable mild stress

The CUMS protocol was performed every day over 7 weeks in which mice were subjected to mild stressors several times per day (Table 1). Some stressors were combined such as: cage tilting and wet sawdust, mice were housed 2 per cage for food and water deprivation, mice were placed 2 per cage for cage tilting, etc. Details of the procedure are described in Supplementary Table 1. All stress manipulations were performed in a room different from the housing room. When no overnight stress was planned, mice were returned to and remained in the housing room.

### Behavioral testing

*Sucrose Preference Test*. Animals were placed in a cage with free access to two pipettes connected to two bottles of water: one contained only water and the other one contained 1% sucrose dissolved in water. The test was conducted during 3 days (d1 to d3). The sucrose solution was freshly prepared each day. To avoid neophobia, sucrose at 1% was presented to all animals for a period of 24 h, a week before the test. During the test, the pipettes and bottles containing sucrose were reversed every 12 h to avoid potential side biases. Sucrose preference was calculated as follow: Sucrose Consumption (mg)/Total Consumption (mg). Preference of CUMS mice was expressed as a percentage of that of control mice.

*Light/Dark Box test*. This apparatus is composed of two compartments: one (2/3 of the box) is directly highly illuminated (820 lm) and the other on (1/3 of the box) is dark. The animal can move freely from one compartment to another. For testing, a single mouse was placed in the left corner of the highly illuminated compartment of the box and the trajectory of the mouse was video recorded and analyzed using A2V Volcano^®^. The time spent in the illuminated side was used as a measure of anxiety-like behavior with avoidance of the brightly lit area being considered a measure of heightened anxiety-like behavior. The test lasted 5 min and the box was cleaned after each trial.

*OpenField test*. Animals were placed in an OpenField maze composed of a wall-enclosed area to prevent the mouse from escaping. For testing, a single mouse was placed at the top left corner, several motor and behavioral activities were video recorded for 5 min, including: time spent in the inner zone, number of entries into the inner zone, duration of grooming, number of fecal boli deposited, latency and number of rearing events (mouse standing on their hind limbs). An increased number of fecal boli, increased rearing, decreased latency of rearing and decreased grooming as well as decreased time spent in the inner zone were used as metrics of increased anxiety-like behavior.

*Odor hedonic value assessment*. Based on work done in mice (Kermen et al., 2016; Midroit et al., 2021), we selected six odorants for this test: three well documented to have positive hedonic values (Limonene + Lim; 5,989-27-5; 0.2% dilution for 1 Pa vapor), Citronellol (Citro; 106-22-9; 17.8% dilution for 1 Pa vapor) and Camphor (Cam; 76-22-2; 0.46% dilution for 1 Pa vapor) and three with well-documented negative hedonic values (Guaiacol (Gua; 90-05-1; 2.08% dilution for 1 Pa vapor), P-cresol (Cre; 6,032-29-7; 1.8% dilution for 1 Pa vapor), and Pyridine (Pyr; 110-86-1; 2% dilution for 1 Pa vapor)). Hedonic preference was defined as approach and avoidance behavior and was validated in reference to investigation times for biologically relevant odors with known aversive or appetitive properties (Kermen et al., 2016). All odorants were diluted on the day of the test in order to have similar vapor pressures (1 Pa; similar perceived intensity) (Mandairon et al., 2006; Moreno et al., 2009).

The test was performed on the one hole-board apparatus as follows: 60 μL of pure odorant was put on cotton batting and placed at the bottom of a pot covered with bedding. The pot was put in the central hole of the board. Mice were placed at the bottom right corner of the board and allowed to freely explore for 2 min. After the 2 min test, the mouse and the pot were removed, and the board was cleaned. The order of odor presentation was randomized. The total time spent exploring the hole (as measured by a photobeam break at the hole entrance) was recorded for each mouse.

### Neurogenesis assessment

*Sacrifice*. To identify populations of cells that were activated in response to a given odorant, mice were odor stimulated for 1 hour with either an unpleasant odorant (Guaiacol; 100 μL of odorant at 1 Pa) or a pleasant odorant (+Limonene; 100 μL of odorant at 1 Pa). Odorants were presented in a tea ball. One hour after the end of odor stimulation, mice were sacrificed by Urethane injection (2 g/kg) followed by intracardiac perfusion of 50 mL of cold fixative (paraformaldehyde 4% in PBS, pH 7.4). Brains were dissected, sunk in sucrose and sectioned (14 μm thick).

*BrdU injections*. Adult-born cells of the OB require several days to migrate from the subventricular zone to the OB, where they differentiate into mature granule and periglomerular cells. To determine the impact of CUMS on integration and differentiation of adult-born cells in the OB, mice were injected with bromodeoxyuridine (BrdU; Sigma; 50 mg/kg in saline three times daily at 2 h intervals, i.p.) 20 d after the beginning of stress procedure. The CUMS procedure continued for several weeks following BrdU injection, with animals being sacrificed at the completion of behavioral testing (38 d after the BrdU injections). This time course of BrdU administration allowed for assessment of CUMS effects on the birth, differentiation, and survival of adult-born cells in the OB during the CUMS procedure.

*BrdU immunocytochemistry*. The protocol has been described in detail previously (Forest et al., 2019). Sections were incubated overnight in a mouse anti-BrdU antibody (1:100, Millipore Bioscience Research Reagents) at 4°C followed by a biotinylated anti-mouse secondary antibody (1:200, Vector Laboratories) for 2 h. The sections were then processed through an avidin-biotin-peroxidase complex (ABC Elite Kit, Vector Laboratories). Following dehydration in graded ethanols, the sections were defatted in xylene and coverslipped in DPX (Fluka, Sigma).

*Double labelling BrdU/cFos immunocytochemistry*. To investigate the density of BrdU-positive cells responding to pleasant and unpleasant odorants, double labelling was performed using mouse anti BrdU (1/100, Merck) and monoclonal rat anti c-Fos (1/2000, Synaptic System) respectively. Appropriate secondary antibodies were used (goat anti-mouse Alexa 546 (1/200, Molecular probes); goat anti-rat Alexa 488 (1/200, Molecular probes) to label cBrdU+/-Fos + cells.

### Data analysis (BrdU and c-Fos levels)

Every fifth coronal section of the olfactory bulb was processed for immunostaining (sampling interval = 70 μm; 3–5 sections per animal were analyzed). Within each analyzed section, every BrdU-positive cell was counted in the granule layer of the right OB using mapping software (Mercator, Explora Nova, La Rochelle, France) coupled to a Zeiss microscope. The mean positive cell density was calculated and averaged within each experimental group.

Fluorescent counting was done using AxioVision (Zeiss) software coupled to a pseudo-confocal Zeiss microscope. We calculated the percentage of BrdU+ cells expressing c-Fos per mouse and averaged the values per group (23 ± 5 cells per animal were assessed). As an additional control, we also counted the density of c-Fos+/BrdU^−^ in the OB (3 sections were analyzed and averaged per animal). All counting was done blind with regards to the identity and treatment group of the animal.

### Statistics

All analyses were performed using Rstudio. For behavioral experiments were used, preliminary tests including Kolmogorov–Smirnov and Levene tests for normality for all data. This resulted in the use of parametric tests: repeated measures analysis of variance (ANOVA) (day as factor) followed by Bonferroni-corrected *post-hoc t*-tests for the sucrose consumption test and two-ways ANOVA (group and hedonic as factors) followed by Bonferroni-corrected *post hoc t*-tests for the olfactory preference test and cellular analysis. For other behavioral tests and analysis of cellular densities, the 2 experimental groups were compared using two sample *t*-tests. Unilateral *t*-tests were used when prior evidence of effects of the CUMS model on depressive-like behavior and anxiety in rodents were reported (Antoniuk et al., 2019; Markov, 2022). The validity of the *t*-test positive results was confirmed using permutation tests. No statistical methods were used to predetermine sample sizes, but our sample sizes were similar to those reported in previous publications (Forest et al., 2019). Data collection and animal assignation to the various experimental groups were randomized and data collected blind to condition.

## Results

### Chronic unpredictable mild stress induced emotional changes in adult mice

**Figure 1:**
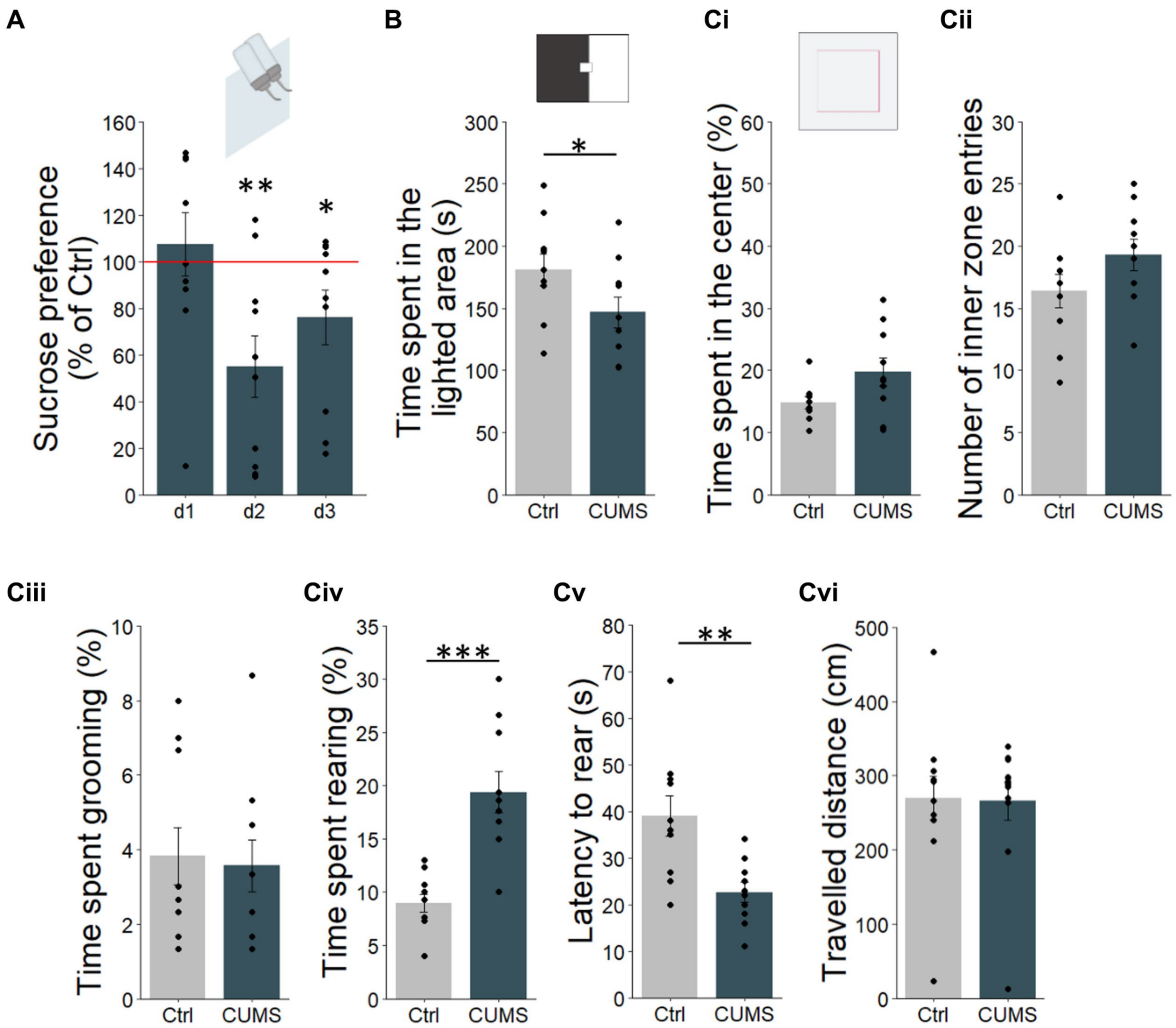
CUMS protocol impacted the mouse emotional behavior. **(A)** The percentage of sucrose consumption was lower at d2 and d3 in CUMS compared to control (Ctrl) mice. **(B)** The CUMS mice showed a significant decrease of time spent in the illuminated compartment compared to the control group in the Light/Dark Box test. **(Ci)** No difference was found between groups in terms of time spent. **(Cii)** nor number of entries in the center of the OpenField apparatus. **(Ciii)** The CUMS mice showed similar levels of grooming to controls, **(Civ)** however they displayed more rearing, **(Cv)** and reared sooner than control mice. **(Cvi)** Locomotion of mice was not altered by CUMS. Points represent individual data means ± s.e.m. Statistical significance depicted as ***p < 0.001, **p < 0.01, and *p < 0.05. *n* = 10/group.

To confirm the effectiveness of the CUMS protocol to alter expression of anxiety and depressive-like behaviors, sucrose preference was assessed in mice over 3 days (Goodwill et al., 2019). The repeated measures ANOVA revealed a day effect on sucrose consumption in CUMS compared to control group (*F*(2,18) = 3.82, *p* = 0.04). More precisely, CUMS mice showed a significant reduction of sucrose consumption at D2 and D3 compared to controls (unilateral one sample *t*-test D1 *p* = 0.6; D2 *p* = 0.006; D3 *p* = 0.03; Figure 1A).

To assess the impact of CUMS on the level of anxiety-like behaviors, mice were tested on two standard behavioral assays, the Light/Dark Box and the OpenField. The Light/Dark Box test relies on the relative time spent exploring each of the two compartments (one illuminated and the other dark). A decrease in time spent in the illuminated compartment is used as a measure of increased of anxiety-like behavior. Results showed that the CUMS group spent less time in the illuminated compartment compared to the control group (unpaired unilateral *t*-test, *p* = 0.02; permutation test, *p* = 0.03, 100,000 permutations; Figure 1B), suggesting a higher level of anxiety in CUMS mice. Similarly, the middle of the OpenField, is generally considered more anxiogenic. Thus, the amount of time that the animal spent in the inner zone (center) of the setup, and the number of entries into center was used as an index of anxiety-like behavior. Here, no significant differences were found between CUMS and control mice (unpaired *t*-test, *p* = 0.96 and *p* = 0.93 respectively; Figures 1Ci,ii). As additional measures, CUMS mice spent a similar amount of time to control animals grooming (unpaired *t*-test, *p* = 0.25; Figure 1Ciii). We observed an increase in time spent rearing (unpaired *t*-test, *p* = 0.005; permutation test, *p* < 0.0001, 100,000 permutations; Figure 1Civ) and a decrease of latency to rear (unpaired *t*-test, *p* = 0.0003; permutation test, *p* = 0.001, 100,000 permutations; Figure 1Cv) in CUMS compared to control mice. To ensure that these effects were not due to changes in locomotor activity, we assessed the total distance travelled by animals in the OpenField for the two groups and found no difference between control and CUMS groups (unpaired *t*-test, *p* = 0.44; Figure 1Cvi). Taken together, these results suggest that CUMS influences multiple aspects of anxiety and depressive-like behavior.

### Chronic unpredictable mild stress induced alterations in odor hedonics

**Figure 2:**
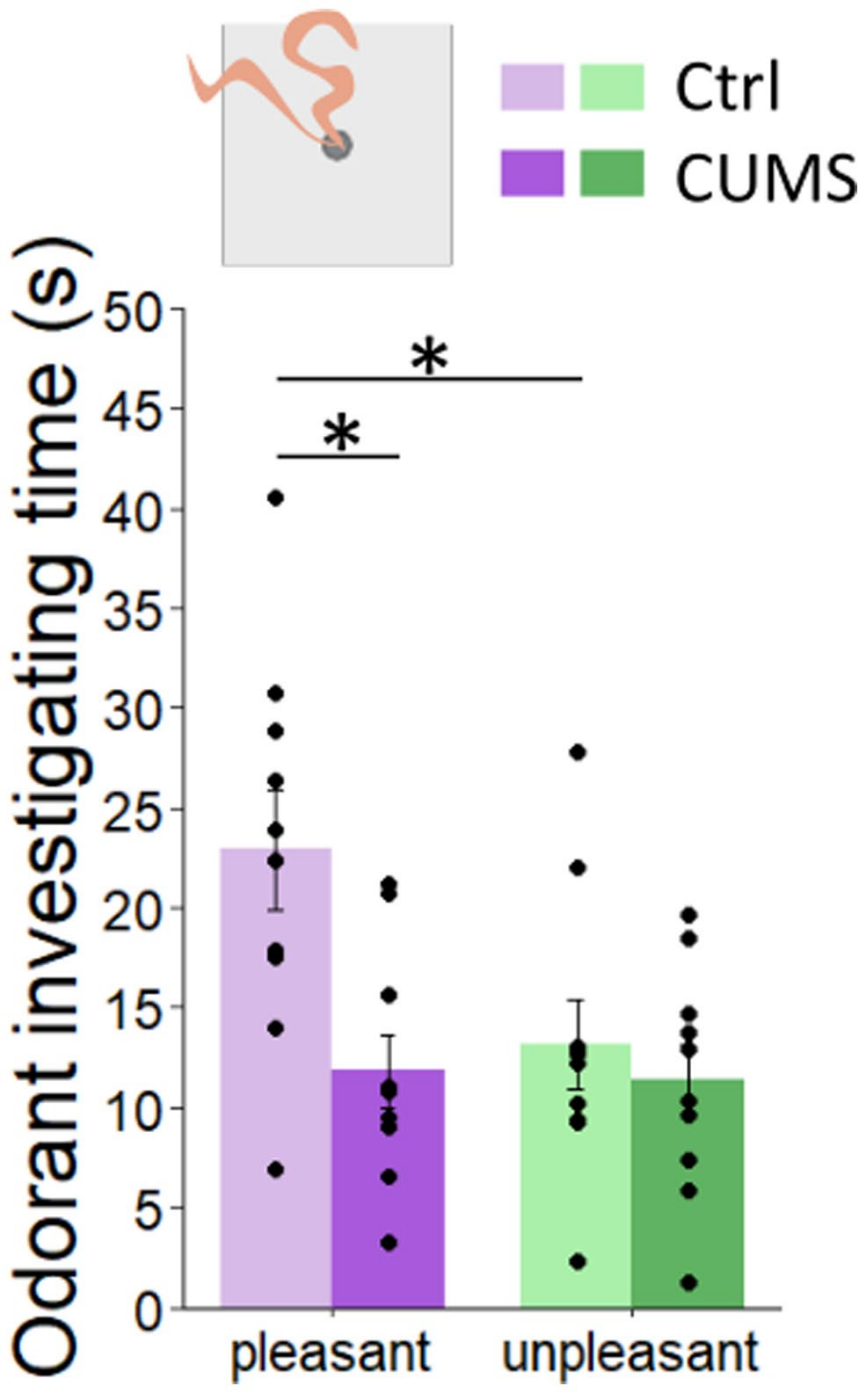
CUMS protocol altered the perception of pleasant odorants. (Top) Experimental set up. (Bottom) (Ctrl) mice spent more time investigating pleasant odorants compared to unpleasant ones. Odor investigation time of the unpleasant odorants was similar between control and CUMS mice while it was reduced for the pleasant odorants in CUMS compared to control group. Points represent individual data means ± s.e.m. Statistical significance depicted as *p < 0.05. *n* = 10/group.

Odor hedonics were automatically assessed using an odor preference test (Mandairon and Linster, 2009), and the time the mouse investigated the odorants was used as an index of odor hedonics (Kermen et al., 2016; Midroit et al., 2021; Figure 2 and Supplementary Figure 1) As expected, investigation time of pleasant odorants was higher compared to unpleasant ones in control mice (2-ways-ANOVA, odor effect: *F*(1.36) = 5.039, *p* = 0.03; Bonferroni *post-hoc* test, *p* = 0.02; Figure 2). Interestingly, we observed a significant effect of experimental group (CUMS versus control: 2-ways-ANOVA; *F*(1.36) = 8.035, *p* = 0.007) and an interaction (*F*(1.36) = 4.2, *p* = 0.04) indicating that the effect of the experimental group depends on the odor hedonic value. In particular, the investigation time of the pleasant odorants was lower in the CUMS group compared to the control one (Bonferroni *post hoc* test, *p* = 0.007; Figure 2) while the investigation time of the unpleasant odorants remained unchanged (Bonferroni *post hoc* test *p* = 0.9; Figure 2).

### Chronic unpredictable mild stress altered adult neurogenesis and reduced the density of adult-born cells responding to pleasant odorants in the OB

**Figure 3:**
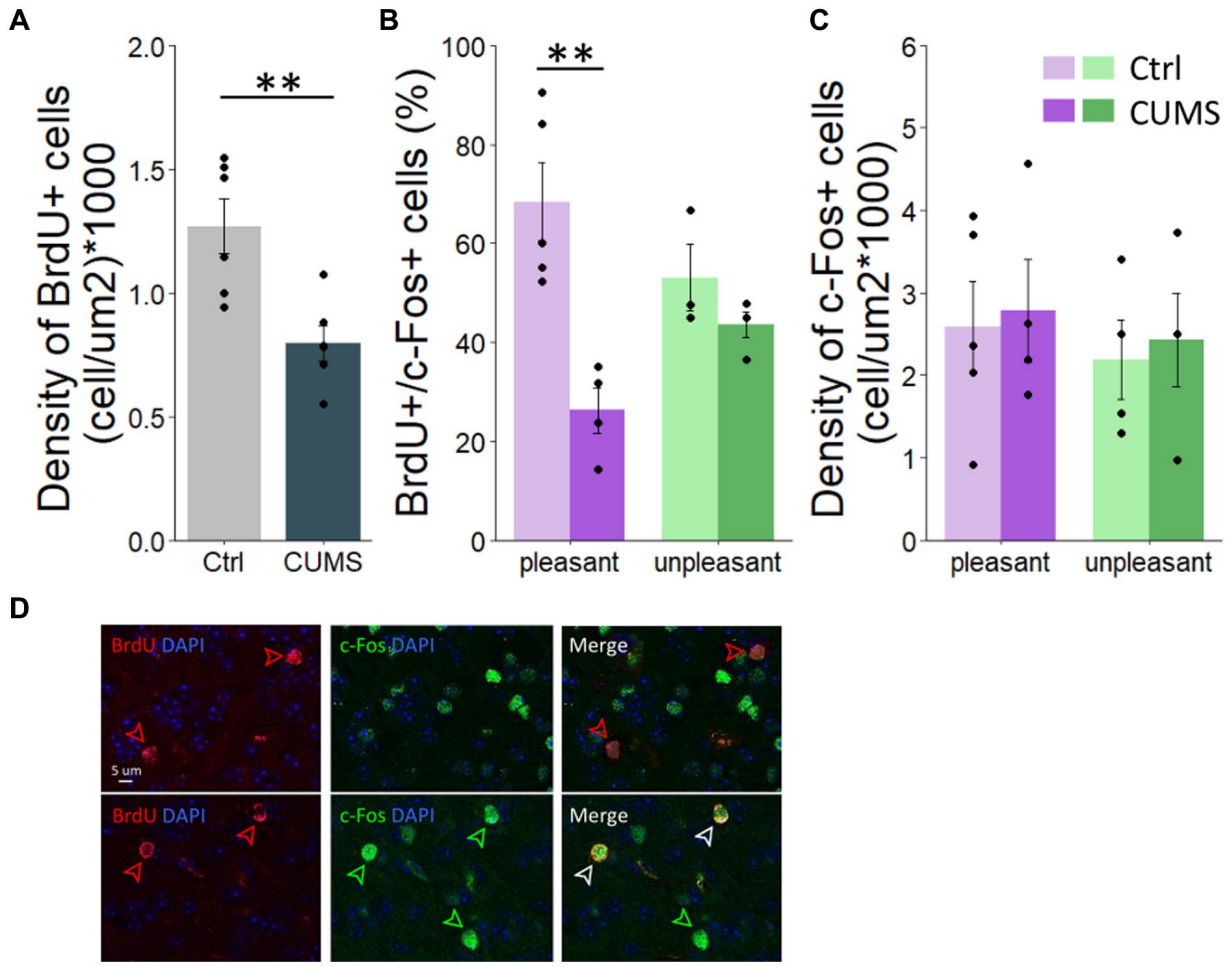
CUMS protocol altered adult-born survival and the activity of adult-neurons involved in pleasant odorant processing. **(A)** The density of BrdU-positive cells was lower in CUMS compared to controls. **(B)** The percentage of BrdU-positive cells responding to pleasant odorants was lower in CUMS mice compared to controls while no difference was found in response to unpleasant odorants. **(C)** The density of c-Fos + cell is similar between groups and hedonic value of odorants. **(D)** Photo of BrdU^+^ cells (red arrows), c-Fos + cell (green arrows) and BrdU+/c-Fos + double-labelled cells (white arrows). Points represent individual data means ± s.e.m. Statistical significance depicted as **p < 0.001. *n* = 5 control and 4 CUMS mice stimulated with pleasant odorants and *n* = 3 control and 4 stressed mice stimulated with unpleasant odorants.

We then tested whether the altered odor hedonic perception after CUMS protocol was associated with changes in olfactory neurogenesis. To do this, we labelled a cohort of adult-born cells integrating in the OB during CUMS protocol by injecting BrdU 20 days after the beginning of the CUMS procedure. This delay allows neuroblasts to migrate from the subventricular zone to the OB and integrate into the pre-existing neural network. We assessed adult-born cell density in the granule cell layer. We found a lower density of BrdU-positive cell in the OB in CUMS compared to control group (unpaired bilateral *t*-test, *p* = 0.006; permutation test, *p* = 0.0043, 924 permutations; Figure 3A). We then assessed the effect of CUMS on the activity of adult-born neurons responding to pleasant versus unpleasant odorants by analyzing c-Fos expression in BrdU labeled cells 1 h following stimulation with either a hedonically pleasant or unpleasant odorant. Using 2-way-ANOVA, we found an effect of experimental group (*F*(1.36) = 19.837, *p* = 0.0007), of odor hedonics (*F*(1.36) = 5.039, *p* = 0.03) and an interaction (*F*(1.36) = 6.634, *p* = 0.02) indicating that the effect of the experimental group depended on the odor hedonics. Specifically, we found that while the percentage of double labelled BrdU/c-Fos cells is not different between the two experimental groups in response to unpleasant odorant (Bonferoni post-hoc test, *p* = 0.3; Figure 3B), a lower percentage of double labelled cells was found in response to pleasant odorant in CUMS compared to control group (Bonferoni post-hoc test, *p* = 0.0002; Figure 3B). This was not due to an overall decrease in neuronal activity in the OB of CUMS animals. Indeed, when assessing the density of c-Fos+/BrdU-labeled cells), a 2-way-ANOVA revealed no effect of experimental group (*F*(1.13) = 0.45, *p* = 0.51), nor of the hedonic value of odorants (*F*(1.13) = 0.972, *p* = 0.7) (Figure 3C). Thus, the decrease in BrdU/c-Fos double labelling reflects less involvement of adult-born neurons in processing pleasant odorants in CUMS group, and not an overall decrease in sensitivity of CUMS animals to pleasant odorants.

## Discussion

In the current experiments, the CUMS paradigm evoked patterns of behavior consistent with increased anxiety and depressive-like behavior and also altered olfactory hedonics. Specifically, our findings confirmed that CUMS induced depressive-like state, with evidence of anhedonia in the sucrose preference test. We also observed an increased anxiety-like behavior in the Light/Dark Box test and the assessment of rearing behavior in the open field. Importantly, we made the novel observation that that CUMS altered odorant hedonics by specifically lowering interest for pleasant odorants. To date, the focus of studies assessing the effect of stress and depression on olfactory perception focus on threshold or intensity (Schablitzky and Pause, 2014). Here, in order to focus on odor hedonics, we chose the strategy of using largely supraliminal concentration of odorants so that the mice, even if they had an altered threshold for odor detection, could still detect the odorant. This is confirmed by the fact that the level of investigation of unpleasant odorants is the same for control and CUMS mice. In addition, simple discrimination is known to be unaffected by chronic stress (Athanassi et al., 2021). While olfactory hedonics is almost never studied in rodent models of stress, several studies in humans have found that pleasant odorants were perceived as less pleasant in patients with depression (anhedonia) and unpleasant ones perceived as more unpleasant (negative alliesthesia) (Atanasova et al., 2010; Naudin et al., 2012; Kazour et al., 2020). In the present study, no difference between control and CUMS groups were found in response to unpleasant odorants suggesting a specific effect of CUMS on the perception of pleasant odorant but a preserved avoidance behavior for unpleasant odorants.

A neural signature of odor hedonics is believed to reside in the granule cell layer of the OB (Kermen et al., 2016). As OB granule cells are born throughout life, we investigated the impact of CUMS on adult neurogenesis. More specifically, we studied the impact of CUMS on the density of adult-born in the OB and their response to odorants to test a possible neural bases for observed effects on olfactory hedonics. First, our results showed that CUMS decreased the density of adult-born neurons in the OB. The lower cell density in the OB could be the result of a reduction of adult-born cell survival in the OB and/or of cell proliferation in the subventricular zone. Here, the time course of labeling was designed to assess aggregate effects on proliferation, integration, and survival of newly born cells, but did not test for selective effects on proliferation. Thus, the contribution of CUMS effects on proliferation versus survival remains open since other studies using various stressors showed different effects on olfactory proliferation (Yang et al., 2011; Belnoue et al., 2013; Siopi et al., 2016; Czarnabay et al., 2019; Wang et al., 2022). The diversity of findings may indicate that the impact of stress on neurogenesis may rely on either discrete (proliferation vs. survival) or synergistic (proliferation and survival) mechanisms in the OB as well as the hippocampus since in the hippocampus, neurogenesis levels and more specifically cell proliferation is altered after stress (corticosterone injections), or CUMS (Guo et al., 2009; Roni and Rahman, 2015; Huang et al., 2019; Li et al., 2021, 2022; Parul et al., 2021). Here, we did not test for changes in hippocampal neurogenesis in our model. While some olfactory associative tasks may involve the hippocampus (Kesner et al., 2011), many of them do not and do not modulate hippocampal neurogenesis (Kaut and Bunsey, 2001; Kaut et al., 2003; Sultan et al., 2010, 2011; Mandairon et al., 2011). Moreover, the olfactory preference task that we use here is a spontaneous olfactory task that do not rely on learning or recall. Thus, even though many studies have found alterations in hippocampal neurogenesis by CUMS, there is no evidence that hippocampal recruitment is involved in olfactory preference testing. Thus it is unlikely that CUMS effects on hippocampal neurogenesis explain olfactory anhedonia.

Interestingly, hippocampal (Huang et al., 2019) and bulbar (Lledo et al., 2006; Hitoshi et al., 2007; Siopi et al., 2016) neurogenesis are restored after anti-depressive treatment. It would be interesting in this context to assess the effect of anti-depressive treatment on olfactory hedonics.

Here, we provided evidence of impaired activity of adult-born granule cells in response to pleasant but not unpleasant odorants after CUMS. Indeed, stressed animals not only displayed lower neurogenesis but also a weaker integration of these cells to the mature network of adult-born granule cells processing pleasant odorants specifically. This suggests that adult born neurons involved in processing pleasant but not unpleasant odorants are not correctly integrated. This suggests a specific sensitivity of OB sub circuits that underlie response to pleasant odorants and could be due to a vulnerability of olfactory sensory neurons recognizing specific molecular features of pleasant odorants and/or a vulnerability of feedback projections to the OB that may modulate survival of newly born cells. This alteration of odor hedonics and bulbar neurogenesis is consistent with recent studies revealing a link between adult-born neuron activity in the hippocampus and hedonic behavior with alleviated depression-like behaviors and an increased resilience against chronic stress when the activity of adult-born neurons is increased (Anacker et al., 2018; Rawat et al., 2022). Finally, granule cells coding for olfactory hedonics regulate mitral cell activity projecting directly to the olfactory tubercle, another key structure of odor hedonics (Midroit et al., 2021). Therefore, any functional alterations of adult-born granule cells in the OB would modulate olfactory processing in the OB and possibly also one of its direct targets, the olfactory tubercle, contributing to odor hedonic impairment.

Olfactory perception and emotional behavior have a bidirectional relationship with olfactory disturbances leading to behavioral profiles indicative of depression-like state in rodents and humans and *vice-versa* (Atanasova et al., 2008; Croy and Hummel, 2017; Taalman et al., 2017; Rochet et al., 2018). Since the OB is tightly linked to the limbic system whose structures are involved in emotional regulation, changes in OB outputs can also modify emotional circuit functioning and behavior (Eiland et al., 2012; Ma et al., 2021). Conversely, alteration of structures of the emotional circuit after stress, like the amygdala, which projects to the OB granule cells (Wen et al., 2019), can lead to change in olfactory processing and perception. Altogether, this may explain why the olfactory perception can be a marker of depression (Croy and Hummel, 2017). Finally, strategies increasing adult hippocampal neurogenesis can alleviate anhedonia (Eliwa et al., 2021), leading to questions about whether a similar mechanism could be employed in the olfactory bulb.

In summary, these represent the first study linking CUMS with altered hedonic processing of odorants, a phenotype that is associated with increased anxiety and depressive-like behavior. We provide novel data indicating that CUMS effects on either integration and activity of adult- born cells may underlie alterations in olfactory hedonics. However, more specific characterization of the activity and interaction of adult-born and pre-existing neurons would be an asset to better understand the mechanistic underpinnings of the altered odor hedonics observed here. Such change in OB activity could contribute significantly to our understanding of the mechanisms of hedonic disturbance of olfactory signals in human population and their contribution to either symptom presentation or worsening quality of life in depressed populations. In addition to enhancing our understanding of the mechanisms underlying impairments in pleasant odor perception in depression, our findings could provide useful information to set up more naturalistic, non-pharmacological approaches to improve olfactory perception, and thereby overall well-being of individuals who have suffered from chronic stress. Indeed, by demonstrating a specific effect of CUMS on positive odor hedonics, future studies could focus on the effects of olfactory training on the hedonic response to odors in order to assess if its effects are mediated by an increase in olfactory hedonic perception. Furthermore, fostering training with consensual pleasant odorants could be more efficient in improving mood disorders in humans, than previous studies using odorants without considering their hedonic value (Umezu, 1999; Ceccarelli et al., 2004; de Almeida et al., 2004; Tokumo et al., 2006; Faturi et al., 2010; Linck et al., 2010; Cryan and Sweeney, 2011; Birte-Antina et al., 2018; Pieniak et al., 2022).

## Data availability statement

The raw data supporting the conclusions of this article will be made available by the authors, without undue reservation.

## Ethics statement

The animal study was reviewed and approved by European Community Council Directive of 22nd September 2010 (2010/63/UE) and the National Ethics Committee (Agreement APAFIS#20702_2019072614086203_v2).

## Author contributions

MB, AD, and NM contributed to conception and design of the study. MB performed the behavioral tests. AA performed the cellular analysis. AA and LC performed the statistical analysis. LC, AA, JB, KB, and NM wrote sections of the manuscript. All authors contributed to the article and approved the submitted version.

## Funding

This work was supported by the CNRS, Inserm, Lyon 1 University (LC), FRM doctoral fellowship (AA) and ENS doctoral fellowship (MB).

## Conflict of interest

The authors declare that the research was conducted in the absence of any commercial or financial relationships that could be construed as a potential conflict of interest.

## Publisher’s note

All claims expressed in this article are solely those of the authors and do not necessarily represent those of their affiliated organizations, or those of the publisher, the editors and the reviewers. Any product that may be evaluated in this article, or claim that may be made by its manufacturer, is not guaranteed or endorsed by the publisher.
