# Supplementary material for: Chronic unpredictable mild stress alters odor hedonics and adult olfactory neurogenesis in mice
Source: Front Neurosci. 2023 Aug 3;17:1224941. doi: 10.3389/fnins.2023.1224941 (PMC10435088; doi:10.3389/fnins.2023.1224941)
Supplement: Supplementary file 1 [file Data_Sheet_1.docx]

**Supplementary materials**

|  | **Week 1** | **Week 2** | **Week 3** |
| --- | --- | --- | --- |
| **D1** | *9.*30am – Cage titled at 45° for 30 minutes  *2pm and 3pm* - Mice hung by the tail for 1 min while sawdust was changed  *5pm* – Lights switched off | *9am* - Cage titled at 45° for 1 hour and lights switched on  *2pm and 3pm* - Mice hung by their tail for 1 min while sawdust bedding was changed and rough handling | *10am* - Cage titled at 45° during 1 hour and light switched on  *2pm* - Sawdust bedding removed and mice hung by their tail for 1 minute prior to pair housing them  *5pm* – sawdust bedding returned and mice hung by tail for 1 minute and then single housed |
| **D2** | *10am* - Sawdust bedding removed and light switched on  *1pm -* Cage titled at 45° for 1 hour  *4pm - S*awdust bedding returned  *6pm -* Mouse cage change and lights kept on | *10.30am* - Removed sawdust bedding and switch-on the light  *11am -* Mouse cage change  *3pm* - Cage titled at 45° for 1 hour  *4 pm* - sawdust bedding returned | *10am, 11am, and 12pm -* Light switched off for 30 minutes  *2pm -* Cage titled at 45° until the next day  *6pm* - Food and water removed and light switched off |
| **D3** | *9.30am –* Mouse cage changed  *2pm -* Mice pair housed in cages with wet sawdust bedding and with lights off | *10am -* Sawdust bedding wetted  *2pm, 3pm, and 4pm -* Lights switched off for 30 minutes  *6pm – Returned to d*ry sawdust bedding and lights remained on | *9.30am* - Food and water put back and lights switched on  *2pm -* Cage titled at 45° for 1 hour |
| **D4** | *10am –* Mice returned to dry sawdust bedding followed by tail hang  *2pm, 3pm, and 4pm -* Light switched off for 30 minutes  *5pm* - Food and water removed with the lights off | *12pm –* Mouse cage change and water and food removed  *2pm -* Switched off the light  *4pm –* Mouse cage change, water and food returned | *10am -* Cage titled at 45° for 1 hour  *2pm -* Mice pair housed with wet sawdust bedding  *5.30pm -* Mice returned to individual housing |
| **D5** | *9.30am –* Water returned and lights switched on  *12pm –* Mouse cage changed  *2pm -* Cage titled at 45° for 1 hour and 30 min  *5 pm* – Mice placed in cage within wet sawdust with food returned while lights are switched off | *10 and 11am -* Mice hung by their tail for 1 min while sawdust bedding was changed  *3 pm –* Mouse cage change | *9.30 am to 4.30 pm –* Lights turned off  *4.30pm -* Mice pair housed in cages with wet sawdust bedding. Tail hang for 1 minute  *4.30pm -* Cage titled at 45° for 1 hour  *5.30pm -* Mice returned to individual housing |
| **D6** | *10am -* Lights turned on  *11am –* Light turned off  *12pm -* Light switched off and mice put back on dry sawdust bedding | *2pm –* Mice placed in cage with wet sawdust bedding | *10am –* Mouse cage change and then hung by their tail for 1 minute  *10,30am –* Mice bedding changed for wet sawdust bedding and lights switched off |
| **D7** | *10am -* Lights Switched off  *11.30am -* Cage titled at 45° for 1 hour and lights switched on | *10am* – Mice returned to dry sawdust bedding and lights switched on  *10.30 and 11.30 am –* Light switched on for 30 minutes | *10am* – Lights switched on, mice returned to dry sawdust bedding, mice hung by the tail for 1 minute  *10.30am and 11.30am -* Light switched off for 30 minutes |

**Supp Table 1:** Detailed example of the CUMS protocol and stressors during 3 weeks.


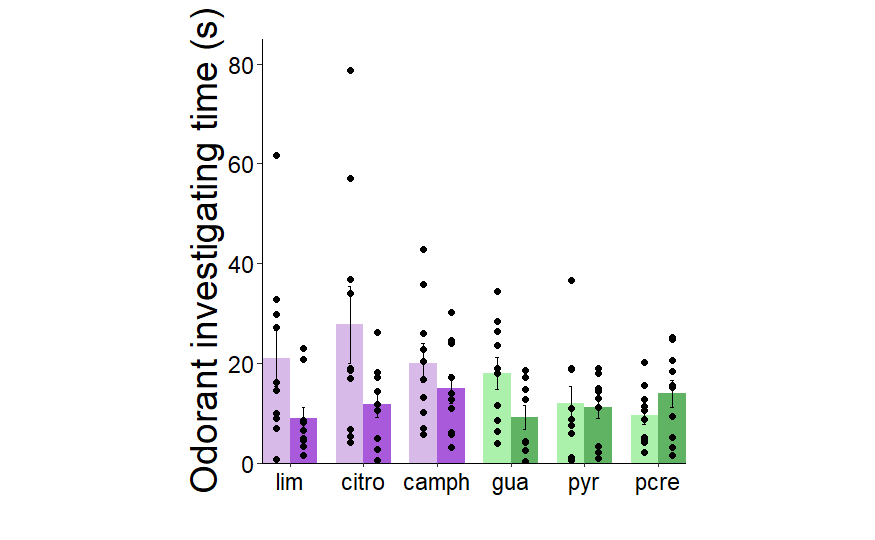


**Supp Fig 1.** Odorant investigation time for the six different odorants: three known to have positive hedonic values (Limonene + (lim), Citronellol (citro) and Camphor (camph) and three with well-documented negative hedonic values (Guaiacol (gua), Pyridine (pyr) and P-cresol (pcre)) in CUMS (dark colors) and control animals (light colors). 2 way ANOVA with condition (CUMS or control) and odor as factors revealed an effect of the condition (F(1.12)=8.79, p=0.003), but no effect of the odor (F(5.21)=1.507, p=0.19) nor interaction between the two (F(5.13)=1.977, p=0.08).
